# Tailoring Compressive Strength and Absorption Energy of Lightweight Multi-Phase AlCuSiFeX (X = Cr, Mn, Zn, Sn) High-Entropy Alloys Processed via Powder Metallurgy

**DOI:** 10.3390/ma14174945

**Published:** 2021-08-30

**Authors:** Ashutosh Sharma, Hansung Lee, Byungmin Ahn

**Affiliations:** 1 Department of Materials Science and Engineering, Ajou University, Suwon 16499, Korea; ashu@ajou.ac.kr; 2 Department of Energy Systems Research, Ajou University, Suwon 16499, Korea; lhssung@ajou.ac.kr

**Keywords:** powder metallurgy, high entropy alloy, lightweight, thermodynamics, absorption energy

## Abstract

The development of lightweight HEAs with high strength and low cost is an urgent requirement. In this study, equimolar AlCuSiFeX (X = Cr, Mn, Zn, Sn) lightweight HEAs were fabricated by advanced powder metallurgy. The mechanical alloying was performed for 45 h, and the powder compacts were densified at 650 °C. The final results revealed that AlCuSiFeSn lightweight HEA was composed of a single face-centered cubic (FCC) and Cu_81_Sn_22_, whereas AlCuSiFeZn showed a dual FCC and body-centered cubic (BCC) structures. Similarly, AlCuSiFeMn alloy contained a BCC + FCC phase with a µ-phase, whereas a σ-phase was present in AlCuSiFeCr in addition to FCC + BCC phases. We also calculated various thermodynamic parameters to predict the solid-solution phase stability of each of the above lightweight HEAs. It was found that lightweight HEAs with additive elements Sn and Zn tend to predominant FCC phases, whereas those with Cr and Mn result in major BCC with hard µ and σ phases, which further improve their mechanical strength. A maximum fracture strain of 23% was obtained for AlCuSiFeSn followed by 19% for AlCuSiFeZn HEA. The compressive fracture mechanisms of these lightweight HEAs are also discussed and reported here.

## 1. Introduction

High-entropy alloys (HEAs) have become increasingly popular since 2004 and are now well recognized [1,2]. Compared with most conventional alloys and steels containing only one principal element, HEAs usually contain five or more principal elements with equiatomic or non-equiatomic compositions varying from 5 to 35 at% [3,4]. All the principal elements in HEAs contribute equally toward their unique properties that may not be attainable in the traditional alloy design approach [5,6].

Most of the initially developed HEAs include the ductile FCC phase Cantor alloy (CrMnFeCoNi) and its derivatives [7]. A number of these HEAs display remarkable properties, such as high strength [7,8,9,10,11], high ductility [12,13], high fracture toughness [14], and exceptional thermal stability [15]. In contrast, BCC Senkov alloy (TiZrHfNbTa) and refractory HEAs are either very hard or brittle [8,9]. Lightweight HEAs in which the density is close to 7 g/cm^3^ have also been actively designed. Youssef et al. designed Al_20_Li_20_Mg_10_Sc_20_Ti_30_ lightweight HEA by cryomilling at subzero temperatures to produce an FCC solid solution phase. After sintering, the bulk lightweight HEA comprised a hexagonal close-packed (HCP) structure with a density of 2.67 g/cm^3^ [16]. Other researchers also produced a lightweight Al_20_Be_20_Fe_10_Si_15_Ti_35_ HEA by casting with a density of 3.91 g/cm^3^ and hardness of −911 HV [17]. However, most of the lightweight HEAs contain toxic, expensive, and scarce elements [18,19,20]. Various strengthening mechanisms, such as solid-solution hardening, grain refinement, precipitation hardening, and heterogeneous structure hardening, have been recommended to balance strength and plasticity in the existing HEAs [21,22,23,24,25]. However, a lightweight HEA with a good combination of mechanical properties remains a challenging problem.

Compared to the popular arc melting methods for production of HEAs, the powder metallurgy route is now increasingly being considered for HEAs [26,27,28]. Al_x_CoCrFeNi HEAs produced by arc melting and sintering of compacted powder show different microstructures (dendritic, particles, or grains) [29]. A similar study showed the formation of different phases in HEAs produced via powder metallurgy and casting [30]. Some of the cast HEAs, such as AlCoCuZnNi [31], TiFeNiCoCu [32], AlCoCrFeNi [33,34], AlCoCrCuFeNi [35,36,37], and CoCrCuFeNiAl0.5 [38], show phase separation behavior with unique microstructural properties, crystal structures, and phase separation due to Cu-rich segregates and ordered L_12_ precipitates [32,35,38].

Powder metallurgy consisting of high-energy ball milling (HEBM) followed by consolidation techniques is widely used to prepare multicomponent alloys and nanomaterials [39]. Advanced spark plasma sintering (SPS) has been widely used to improve the densification rate of resultant bulk HEAs [40]. Recently, equiatomic non-Cantor AlCuSiFeZn HEA produced by HEBM and SPS exhibited a combination of FCC and BCC type solid solutions [41]. Due to the continuous advancement of HEAs in various fields, it is important to understand the various new elemental additions in HEAs. Therefore, this study explores the phase evolution and stability in the processing of AlCuSiFe (Zn, Sn, Cr, Mn) HEAs via HEBM and SPS. We selected (i) Al, Cu, Si, and Fe; and (ii) Cr (BCC), Mn (BCC), Zn (hexagonal close packed, HCP), and Sn (body centered tetragonal, BCT) to prepare a series of lightweight, low cost, and high strength AlCuSiFe (Cr, Mn, Zn, Sn) HEAs. The potential applications of light weight multi-phase AlCuSiFeX (X = Cr, Mn, Zn, Sn) high entropy alloys include in automotive engine parts, heat exchangers, pipelines and boilers, rocket nozzles, high temperature turbine blades, load bearing components in bridges, and the transportation industry and energy sectors. Lightweight HEAs enable the reduction of both energy consumption and the harmful impact of toxic carbon emissions on the environment. Finally, the microstructure and compressive properties of the HEA alloy systems were systematically investigated.

## 2. Materials and Methods

The elemental powders for the production of lightweight HEAs were Al, Cu, and Sn, which were 99.9% pure, whereas Si, Fe, Mn, Cr, and Zn were 99.8% pure. These powders in four sets of equiatomic compositions, AlCuSiFe-x (x = Zn, Sn, Cr, Mn) were HEBMed using a Retsch PM-400 planetary ball mill. The milling media included hardened stainless-steel vial and ball sets. Here, the ball-powder weight ratio was maintained at 10:1 and milling was performed for 45 h. Stearic acid (0.2 wt%) was added to the powder mixture to avoid cold welding of the powders.

The HEBMed powders were densified at 50 MPa and 650 °C using an SPS machine (Dr. Sinter LAB Jr. SPS, Saitama, Japan). The SPS cycle consisted of a heating rate of 100 °C/min to 600 °C and a subsequent rise to 650 °C at 50 °C/min covering a total span of 8 min. The final shape of the product was a compact cylinder (Ø 20 mm × 6 mm). The calculated theoretical densities of lightweight HEAs with x = Cr, Mn, Zn, and Sn were 5.20, 5.23, 5.28, and 5.58 g/cm^3^, respectively.

The microstructural characterization of the HEBMed and SPSed samples was performed using a field emission scanning electron microscope (FESEM, JEOL JSM 7500F). The compositional analysis was performed by energy-dispersive X-ray spectroscopy (EDS). To identify the phase structure and evolution, we used a D8 ADVANCE X-ray diffractometer (XRD, λ = 1.540598 Å) operated at 40 kV and 40 mA, where the samples were scanned from 20° to 80° in 0.02° steps.

For compressive strength evaluation, we micromachined the compacts into cylindrical shapes (Ø 3 mm × 6 mm). The measurements were carried out on a universal testing machine (Instron 5569 UTM) at a loading rate of 0.1%/s. The compressive fracture and yield stress and absorption energy were determined from the stress–strain diagram. A maximum of five readings were obtained and averaged.

## 3. Results and Discussion

### 3.1. Powder Alloying

**Figure 1:**
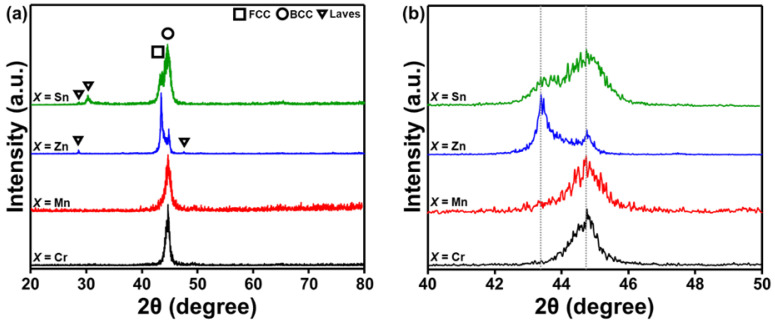
(**a**) XRD patterns of the HEBMed AlCuSiFeX (X = Cr, Mn, Zn, Sn) HEAs after 45 h and (**b**) the magnified major peak of (**a**).

The XRD patterns of the AlCuSiFeX (X = Cr, Mn, Zn, Sn) HEAs are shown in Figure 1. The various phases were marked in the XRD patterns. The predominant peaks in the XRD patterns indicate the presence of FCC and BCC solid solutions (Figure 1a). The grinding process occurred separately because of the difference in density and diffusion kinetics of each element during milling. However, up to a certain milling period (45 h), the grinding process was homogenized due to the increased alloying. A single BCC solid solution phase was formed in AlCuSiFeCr and AlCuSiFeMn HEAs. A dual FCC + BCC solid-solution phase existed in AlCuSiFeZn and AlCuSiFeSn HEAs with the formation of Laves phases. After the inspection of the XRD patterns at higher resolution (Figure 1b), the evolution of dual phase FCC + BCC solid solution phases was conformed. A higher broadening of the peaks in these HEAs indicated the nanocrystallinity of the phases after 45 h of HEBM.

According to the study by Guo et al., the FCC phase is stable when valence electron concentration (*VEC*) ≥ 8; the BCC phase is stable when *VEC* ≤ 6.87; and BCC + FCC phases co-exist when 6.87 ≤ *VEC* ≤ 8 [42]. It can be seen that the *VEC* criteria (*VEC* for AlCuSiFeCr: 6.4, AlCuSiFeMn: 6.6, AlCuSiFeZn: 7.6, AlCuSiFeSn: 8) are weaker in AlCuSiFeX (X = Cr, Mn), whereas the microstructure is retained in AlCuSiFeX (X = Zn, Sn). The formation of metastable Laves phases in AlCuSiFeX (X = Zn, Sn) HEAs was observed. Metastable phases have been observed to form during the HEBM due to the insufficient milling energy available to homogenize the milled powder from the non-equilibrium state [26,43].

### 3.2. Spark Plasma Sintered Pellets

**Figure 2:**
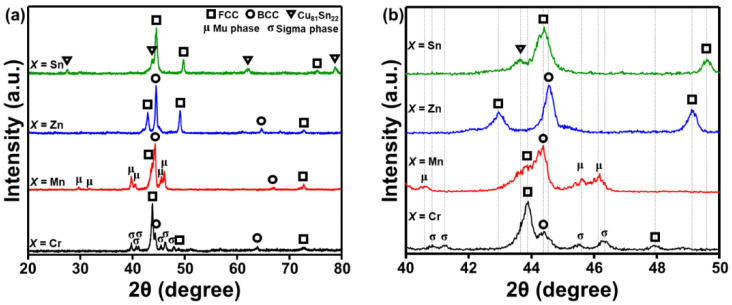
(**a**) XRD analysis of the SPSed AlCuSiFeX (X = Cr, Mn, Zn, Sn) HEAs and (**b**) magnified view of (**a**).

Figure 2 shows the XRD patterns of the SPSed AlCuSiFeX (X = Cr, Mn, Zn, Sn) HEAs at 650 °C. The various crystalline phases are FCC, BCC, µ-, and σ-phases, and Cu_81_Sn_22_ (Figure 2a). The dominant phases are FCC + BCC + σ-phase, FCC + BCC + µ-phase, FCC + BCC, and FCC + BCC + Cu_81_Sn_22_ phase in AlCuSiFeX (X = Cr, Mn, Zn, Sn), respectively. In addition, the Laves phases in AlCuSiFeZn disappeared after HEBM. Table 1 summarizes the various phases formed during HEBM and SPS for comparison. Laves phases disappeared in AlCuSiFeSn and a new Cu-Sn IMC evolved. This may be due to the diffusion of Cu and Sn atoms during SPS. Due to the high ductility of Sn and its limited solubility in other elements, the segregation of Cu-Sn IMCs is expected in the high entropy system. A similar behavior was also observed by Liu et al. in FeCoCuNiSnx HEAs [44], where the presence of Cu_81_Sn_22_ compound was noticed when Sn content > 0.05%.

As mentioned previously, the *VEC* of HEAs in the present case falls below 6.87, for AlCuSiFeCr and AlCuSiFeMn, and thus, the BCC phase is expected. The *VEC* dual FCC + BCC phase is expected for AlCuSiFeZn, whereas a single FCC phase is expected for AlCuSiFeSn. However, after SPS, the formation of the µ-phase is justified in AlCuSiFeMn, similar to the report of Wu et al. [45]. Metastable σ- and µ-phases were formed in AlCuSiFeCr and AlCuSiFeMn HEAs. This result is similar to those reports where HEAs containing Cr and Mn indicate σ- and µ-phase formation after SPS [45,46,47]. It should be noted that HEBM results in the formation of metastable phases that often transform or become stable after SPS. Enhanced enthalpy of nanopowders during HEBM lowers the driving force for the change in phase transformation during SPS [40]. The metastable phases produced during HEBM induce realignment of the grain boundaries and disappearance of crystal defects leads to the formation of new stable phases [40,43].

The density of the investigated HEAs falls within the range of common lightweight alloys. According to the rule of mixture, the density of the investigated HEAs AlCuSiFeCr, AlCuSiFeMn, AlCuSiFeSn, and AlCuSiFeZn HEAS were 5.2, 5.25, 5.58, and 5.28 g/cm^3^, respectively. These values are almost comparable and are in close proximity to those of conventional titanium alloys (4.51–5.75 g/cm^3^).

### 3.3. Microstructure

**Figure 3:**
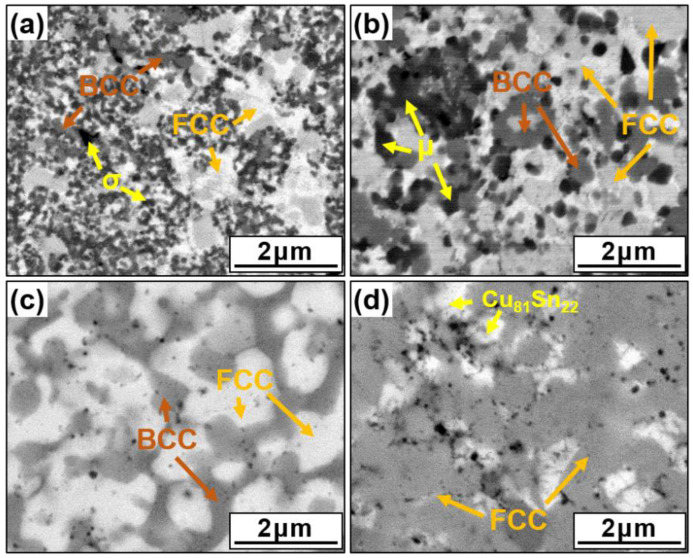
SEM images of the SPSed HEAs. (**a**) AlCuSiFeCr, (**b**) AlCuSiFeMn, (**c**) AlCuSiFeZn, and (**d**) AlCuSiFeSn.

Figure 3 presents SEM images of the various lightweight AlCuSiFeX (X = Cr, Mn, Zn, Sn) HEAs. Typical equiaxed grain structures were observed in all sintered samples. The HEA matrix was divided into different contrast regions (light and dark), which appeared prominently in the matrix. The results of detailed EDS mapping analysis are shown in Table 2, which indicates the composition of the light- and dark-colored regions. The dark BCC area corresponds to the Al-Fe-Si phase with 25–27 at% Al, and the light FCC area corresponds to the Cu-Al-rich phase. The Cr-rich σ-phase in AlCuSiFeCr and the Si-rich µ-phase in AlCuSiFeMn were observed (Figure 3a,b). For AlCuSiFeCr, the Cr-rich σ phase results from the decomposition of the BCC lattice. A similar observation with different BCC structures was noted for Al_x_CoCrCoFeNi (x = 0–3.0) [35,38]. According to the diffraction peaks, the µ-phase was formed in the AlCuSiFeMn HEA. The formation of the µ-phase was observed in other reports on Mn-containing HEAs [45]. The AlCuSiFeZn HEA was composed of FCC and BCC solid solutions, whereas AlCuSiFeSn had a single FCC phase with Cu_81_Sn_22_ (Figure 3c,d), which was also confirmed by XRD results.

The elemental compositions given in Table 2 indicate Cu-rich FCC and Si-rich BCC phases, and that Cu undergoes segregation during SPS. Therefore, the Cu atomic fraction in the FCC phase increases to 34 at%, for x = Zn, whereas in Cu_81_Sn_22_, it increases to over 65 at% for x = Sn (Table 2). The interactions among various elemental segregation and separation behaviors can be identified from the mixing enthalpies of the binary alloy constituents (Table 3).

The mixing enthalpies between the binary pairs of Cu and Al, Fe, Si, Cr, Mn, Zn, and Sn are −1, 13, −19, 12, 4, 1, and 7 kJ/mol, respectively. Similarly, the mixing enthalpies between Al and Cu, Si, Fe, Cr, Mn, Zn, and Sn are −1, −19, −11, −10, −19, 1, and 4 kJ/mol, respectively. This means that Al attracts Fe, Si, Mn, and Cr to the BCC area. Due to the positive mixing enthalpy of Cu-Zn (1 kJ/mol), Cu-Sn (7 kJ/mol), Cu-Cr (12 kJ/mol), and Cu-Mn (4 kJ/mol), these are segregated toward the FCC region. However, the high negative mixing enthalpies of Al-Si (−19 kJ/mol) and Al-Fe (−11 kJ/mol) attract Fe and Si to the BCC regions.

### 3.4. Thermodynamic Analysis

The thermodynamic parameters of HEAs, such as the atomic size difference (*δ*), mixing enthalpy (Δ*H_mix_*), mixing entropy (Δ*S_mix_*), electronegativity difference (Δ*χ*), and valence electron concentration (*VEC*), are determined as follows [49,50,51,52]:

(1) $$\delta =\sqrt{\overset{n}{\underset{i=1}{\sum }}c_{i}{\left(1-\frac{r_{i}}{r}\right)}^{2}}\times 100$$

(2) $$\Delta H_{mix}=4\;\overset{n}{\underset{i=1,\;\;i\neq j}{\sum }}\Delta H_{ij}^{mix}c_{i}c_{j\;\;\;\;\;\;\;\;\;\;}$$

(3) $$\Delta S_{mix}=-R\overset{n}{\underset{i=1}{\sum }}c_{i\;}ln\;c_{i\;}$$

(4) $$\Delta \chi =\sqrt{\overset{n}{\underset{i=1}{\sum }}c_{i}{\left(\chi_{i\;}-\chi \right)}^{2}}\;$$

(5) $$VEC=\overset{n}{\underset{i=1}{\sum }}c_{i\;}VEC_{i\;}$$

where *n* = 5, *c_i_* and *c_j_* indicate concentration in at% of the *i*th and *j*th elements, respectively, and *r_i_* = atomic size of the ith element. *r* and *χ* are the mean atomic size and electronegativity of all the elements, respectively. Δ*H_mix_*_, *ij*_ is the mixing enthalpy of equiatomic (*i*, *j*) alloys. *R* = 8.314 J/mol·K is the universal gas constant. Yang’s interaction parameter is given by:

(6) $$\Omega =\frac{T_{m\;}\Delta S_{mix}}{\left|\Delta H_{mix}\right|}$$

(7) $$T_{m\;}=\overset{n}{\underset{i=1}{\sum }}c_{i}{\left(T_{m}\right)}_{i}$$

For phase stability, Yang’s interaction parameter *Ω* ≥ 1.1 with *δ* ≤ 6.6%; Tm is the mean temperature of the HEA. Wang et al. [53] defined another interaction parameter similar to that of Yang [49] for equiatomic HEAs, given by:

(8) $$\varphi \geq \frac{1.1}{k_{n}}=\varphi_{n\;}\;;\;\;\;\;\Omega =\frac{k_{n}T_{tot}}{\left|H_{tot}\right|}=k_{n}\varphi$$

(9) $$\Delta H_{mix}=4\frac{H_{tot}}{n^{2}}$$

(10) $$H_{tot}=\overset{n}{\underset{i=1,i\neq j}{\sum }}\Delta H_{ij}^{mix}$$

**Figure 4:**
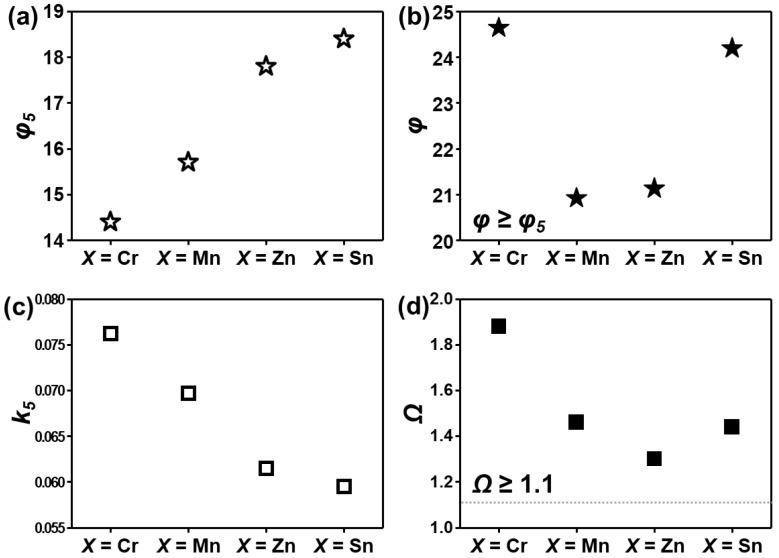
Various thermodynamic parameters (**a**–**d**) calculated for AlCuSiFeX (X = Cr, Mn, Zn, Sn) HEAs.

*H_tot_* is the total enthalpy of binary pairs of individual elements and *n* is the number of elements. The coefficient *k* is a function of *n*. For *n* = 5, *k*_5_ is fixed. *T_tot_* is the sum of elemental melting points of the HEA ($\overset{n}{\underset{i=1}{\sum }}{\left(T_{m}\right)}_{i}$. The various thermodynamic parameters (*φ*, *φ*_5_, *k*_5_, *Ω*) were estimated. The parameters for AlCuSiFeX (X = Cr, Mn, Zn, Sn) are summarized in Figure 4a–d. Inspection of Figure 4a–b shows that the parameter *φ* ≥ *φ*_5_ for all X (X = Cr, Mn, Zn, Sn) favors solid-solution formation. This observation is consistent with the results of Wang [53].

In this study, for five component alloys, *φ* ≥ *φ*_5_ = 14.3 favors the formation of solid solution and theoretically *k*_5_ = 0.057–0.076 (Figure 4c). However, the presence of the µ-phase in AlCuSiFeMn and the σ-phase in AlCuSiFeCr HEAs shows that Yang’s interaction parameter is a necessary condition but not essential for the stable solid-solution phase. Additionally, it can be verified by the interaction parameter that Cr- and Mn-containing HEAs satisfy the criterion of the *Ω* parameter (Figure 4d). According to the phase-formation map of *Ω* and *δ* for the HEAs given by Yang [49], large values of the *Ω* parameter and a large atomic size mismatch cause severe lattice distortion and precipitation of ordered compounds is observed. It is noteworthy that the *VEC* rule does not hold well when the HEA is composed of immiscible elements. In this case, the different elements mix and then separate from one another, and the effect of *VEC* may be significantly weaker [52]. Such contradictions may also be correlated to the different processing conditions compared with those of Guo [42], who studied cast HEAs.

### 3.5. Mechanical Properties

**Figure 5:**
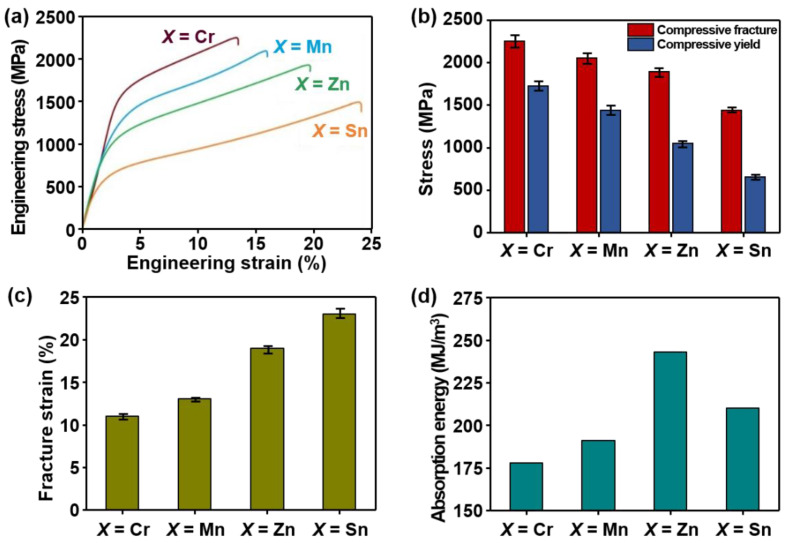
(**a**) Engineering stress–strain curve, (**b**) compressive fracture and yield stress, (**c**) fracture strain, and (**d**) absorption energy recorded from stress strain curves for AlCuSiFeX (X = Cr, Mn, Zn, Sn) HEAs.

The microhardness and various compressive properties, for example, YS, compressive fracture and yield stress, fracture strain, and absorption energies recorded for lightweight AlCuSiFeX (X = Cr, Mn, Zn, Sn) HEAs are shown in Figure 5a–d. The engineering stress and strain curves are also displayed in Figure 5a.

It can be seen that AlCuSiFeSn- and AlCuSiFeZn-based lightweight HEAs exhibit a high plastic region compared to AlCuSiFeCr and AlCuSiFeMn HEAs (Figure 5a). Similarly, the compressive yield stress of the AlCuSiFeX HEAs was in the range of 650–1600 MPa. The compressive fracture stress of the AlCuSiFeX HEAs was in the range of 1350–2100 MPa (Figure 5b). The maximum compressive fracture and yield stress were obtained for the AlCuSiFeCr HEA at 2080 MPa and 1570 MPa, respectively. A maximum elongation of 23% was obtained for AlCuSiFeSn, followed by 19% for AlCuSiFeZn (Figure 5c). The results show that the addition of Zn and Sn, compared with those of Cr and Mn, significantly enhanced the elongation. In contrast, the ultimate strain was minimal for AlCuSiFeCr and AlCuSiFeMn. Similarly, the absorption energies of these HEAs follows the trend in the order AlCuSiFeZn > AlCuSiFeSn > AlCuSiFeMn > AlCuSiFeCr. The absorption energy of AlCuSiFeSn (215 MJ/m^3^) is slightly lower than AlCuSiFeZn (245 MJ/m^3^). This can be due to the presence of Cu_81_Sn_22_ IMCs. In contrast, the absorption energies of AlCuSiFeCr (176 MJ/m^3^) and AlCuSiFeMn (188 MJ/m^3^) were minimal. This can be ascribed to the presence of harder phases in the HEAs, such as brittle µ- and σ-phases, which increase the strength at the cost of ductility.

The increased solution hardening effect in such dual-phase HEAs may be due to the difference in coordination number (12 for FCC and 8 for BCC), which leads to a larger fraction of atomic pairs with dissimilar atoms [2]. The high ductility of FCC-based alloys can be rationalized using the Hollomon equation [54,55]:

(11) $$\sigma =K\varepsilon^{n},\;\mathrm{where}\;n\;\mathrm{can}\;\mathrm{be}\;\mathrm{expressed}\;\mathrm{as}\;n=d\left(\frac{ln\sigma }{ln\varepsilon }\right)$$

Differentiating (11), the Crussard–Jaoul (C–J) equation can be used to study the hardening behavior:

(12) $$ln\left(\frac{d\sigma }{d\varepsilon }\right)=\ln\left(Kn\right)+\left(n-1\right)ln\varepsilon$$

The Swift model presents the modified (C–J) equation according to the strain and stress equation as follows:

(13) $$\varepsilon =\varepsilon^{0}+c\sigma^{m}$$

where *ε*^0^ and *c* are the material constants. The value of *m* can be extracted by differentiating Equation (11):

(14) $$ln\left(\frac{d\sigma }{d\varepsilon }\right)=\left(1-m\right)ln\sigma -lncm$$

In Equations (11)–(14), we can see that the strain hardening increases with increasing strain, i.e., for each case with slopes (*n*, *n* − 1, 1 − *m*). The hardening mechanism of such HEAs proceeds in two steps. Initially, at smaller strains, the deformation is mild from plastic (FCC) to mixed ductile-brittle deformation (FCC, BCC). The FCC phase carries most of the load and transfers it to the other secondary phases (BCC, precipitates). Furthermore, at higher strains, deformation of both FCC and BCC, and elastic deformation of precipitates take place, thereby creating a high density of geometrically necessary dislocations to improve the strain hardening. This behavior is similar to that of dual-phase steels with a soft ferrite phase and a hard martensite phase [55,56].

### 3.6. Fracture Analysis

**Figure 6:**
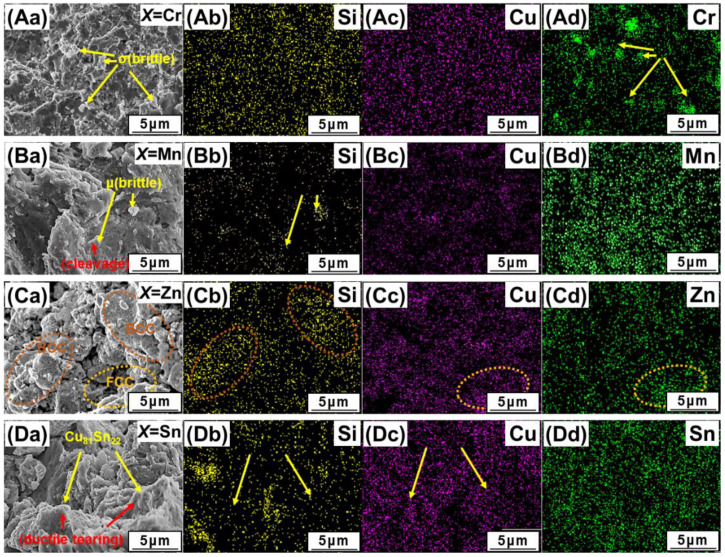
SEM-EDS study of the compressive fracture surfaces of (**Aa**) AlCuSiFeCr, (**Ba**) AlCuSiFeMn, (**Ca**) AlCuSiFeZn, (**Da**) AlCuSiFeSn HEAs. The corresponding EDS maps are shown in (**Ab**–**Ad**,**Bb**–**Bd**,**Cb**–**Cd**,**Db**–**Dd**).

The fracture morphologies and EDS mapping analysis of the compressive-tested samples are shown in Figure 6. We can see that the fractured surface of AlCuSiFeCr HEAs is quite smooth with a brittle Cr-rich σ-phase indicating a brittle surface cleavage plane (Figure 6Aa). The fractured surface showed Cr-rich pits during failure (Figure 6Ad, marked with yellow arrows). The SEM image shows that the Si-Cr-rich debris has a sharp faceted structure. In contrast, the fracture surface of AlCuSiFeMn has some powdery loose Mn-Si-rich debris and fine microcracks, showing a mixed ductile brittle failure (Figure 6Ba–Bd). The debris shows two types of compositions, namely, the Si-rich and Mn-rich µ-phase. The presence of various microcracks that may have been generated during the compressive crushing of the brittle µ-phase represents an intergranular failure of the HEA (Figure 6Bb, marked with yellow arrows).

There are several globular flakes and smooth regions created in AlCuSiFeZn during the evaporation and degassing of Zn. There is also a signficant presence of Cu and Zn in the globular flakes (encircled regions), which are engraved by hard Si-rich phases, and the smooth surface is rich in Si (Figure 6Ca–Cd). Some chipped out regions are also present, indicating a mixed ductile type failure. The composition of this chipped region was high in Zn indicating intergranular fracture.

Similarly, Cu-rich particles and Si-Cu-Sn facets were formed in AlCuSiFeSn HEAs. A mixed brittle and ductile type failure, with a number of rough quasi-cleavage planes and slip lines, were observed in the AlCuSiFeSn HEA (Figure 6Db,Dc, marked with yellow arrows), indicating a typical cleavage fracture and slip formation. The slip formation is generally associated with the plastically deformed FCC phase separated by a mild ductility Cu_81_Sn_22_ phase. The cleavage facets are rich in Si. This type of mixed ductile and brittle failure was also previously observed in Si-based HEAs [41]. Therefore, the overall deformation mechanism of AlCuSiFeX (X = Cr, Mn, Zn, Sn) HEAs comprises loose debris generation, faceted cleavage formation, and slip separation of the phases.

## 4. Conclusions

We prepared a set of lightweight multi-phase HEAs, AlCuSiFeX (X = Cr, Mn, Zn, Sn) by HEBM followed by SPS. The microstructural observations showed that the AlCuSiFeCr alloy consisted of a dominant BCC phase and a minor FCC phase with a σ-phase, whereas AlCuSiFeMn consisted of a dominant BCC phase and a minor FCC phase with a µ-phase. Similarly, AlCuSnFeZn showed dual BCC and FCC phases, whereas AlCuSiFeSn showed a single FCC phase with Cu_81_Sn_22_. It was shown that the mixing enthalpies of individual binary alloys play a key role in phase-formation and separation behavior in these HEAs. The thermodynamic analysis shows that our results also conform to the interaction parameters for equiatomic alloys proposed by Yang and Wang for the phase stability of equiatomic HEAs.

Compressive studies show that the values for the compressive yield and fracture stress, and fracture strain of the AlCuSiFeX (X = Cr, Mn, Zn, Sn), follow the order of X = Cr > Mn > Zn > Sn. However, the fracture strain of these HEAs shows the reverse trend. The maximum elongation (23%) was observed in AlCuSiFeSn, followed by 19% in AlCuSiFeZn. The absorption energy of these lightweight AlCuSiFeX (X = Cr, Mn, Zn, Sn) HEAs follows the order of X = Zn > Sn > Mn > Cr. The mechanism of failure of the AlCuSiFeX (X = Cr, Mn, Zn, Sn) HEAs depends upon the phase-formation behavior in HEAs. A failure mechanism of cleavage fracture and crack formation was present in AlCuSiFeCr, whereas loose powder debris type failure was evident in AlCuSiFeMn. In AlCuSiFeZn and AlCuSiFeSn, mixed ductile and brittle fractures with quasi-cleavage fracture and slip formation were observed.

## Figures and Tables

**Table 1:** Phase evolution after HEBM and SPS from XRD analysis.

Composition	Phases	
	After HEBM (45 h)	After SPS (650 °C)
AlSiFeCuCr	BCC	BCC/FCC/σ
AlSiFeCuMn	BCC	BCC/FCC/μ
AlSiFeCuZn	BCC/FCC/Laves	BCC/FCC
AlSiFeCuSn	BCC/FCC/Laves	FCC/Cu_81_Sn_22_

**Table 2:** EDS compositional data of the AlSiFeCuX (X = Cr, Mn, Zn, Sn) alloys in at%.

HEA	Composition	Al	Si	Fe	Cu	X
AlSiFeCuCr	Nominal	20	20	20	20	20
	Actual	18.23	16.22	23.32	19.58	22.65
	FCC	22.67	13.45	15.23	26.72	21.93
	BCC	27.00	24.60	15.05	10.20	23.15
	σ	21.66	3.27	14.44	6.28	54.35
AlSiFeCuMn	Nominal	20	20	20	20	20
	Actual	18.96	16.27	16.85	28.06	19.86
	FCC	18.71	17.16	15.13	26.64	20.36
	BCC	27.26	26.33	14.62	7.42	14.37
	μ	35.23	37.32	6.61	4.68	17.16
AlSiFeCuZn	Nominal	20	20	20	20	20
	Actual	18.83	21.09	19.88	19.29	20.91
	FCC	10.65	11.58	19.00	33.95	24.82
	BCC	25.53	25.21	19.70	16.50	13.06
AlSiFeCuSn	Nominal	20	20	20	20	20
	Actual	24.16	16.95	17.24	25.76	15.89
	FCC	29.59	24.35	17.02	15.11	14.93
	Cu_81_Sn_22_	5.23	4.76	8.75	65.07	16.19

**Table 3:** Enthalpy of mixing of binary pairs (*i*, *j*) of individual elements (kJ/mol) of the constituent binary equiatomic HEAs [48].

	Al	Cu	Si	Fe	Cr	Mn	Zn	Sn
**Al**	0	−1	−19	−11	−10	−19	1	4
**Cu**	−1	0	−19	13	12	4	1	7
**Si**	−19	−19	0	−35	−37	−39	−18	6
**Fe**	−11	13	−35	0	−1	0	4	11
**Cr**	−10	12	−37	−1	0	2	5	10
**Mn**	−19	4	−39	0	2	0	−6	−7
**Zn**	1	1	−18	4	5	−6	0	1
**Sn**	4	7	6	11	10	−7	1	0

## Data Availability

The data required to reproduce these findings cannot be shared at this time, as the research data are confidential.
